# Strong Light–Matter Coupling in Lead Halide Perovskite Quantum Dot Solids

**DOI:** 10.1021/acsnano.3c10358

**Published:** 2024-02-01

**Authors:** Clara Bujalance, Laura Caliò, Dmitry N. Dirin, David O. Tiede, Juan F. Galisteo-López, Johannes Feist, Francisco J. García-Vidal, Maksym V. Kovalenko, Hernán Míguez

**Affiliations:** ‡ Multifunctional Optical Materials Group, Institute of Materials Science of Sevilla, Consejo Superior de Investigaciones Científicas − Universidad de Sevilla (CSIC-US), Américo Vespucio 49, Sevilla 41092, Spain; § Laboratory of Inorganic Chemistry, Department of Chemistry and Applied Biosciences, ETH Zürich, Zürich CH-8093, Switzerland; ⊥ EMPA − Swiss Federal Laboratories for Materials Science and Technology, Dübendorf CH-8600, Switzerland; || Departamento de Física Teórica de la Materia Condensada and Condensed Matter Physics Center (IFIMAC), Universidad Autónoma de Madrid, Madrid 28049, Spain

**Keywords:** quantum dot solids, perovskites, strong exciton-photon coupling, polaritons, optical microcavities

## Abstract

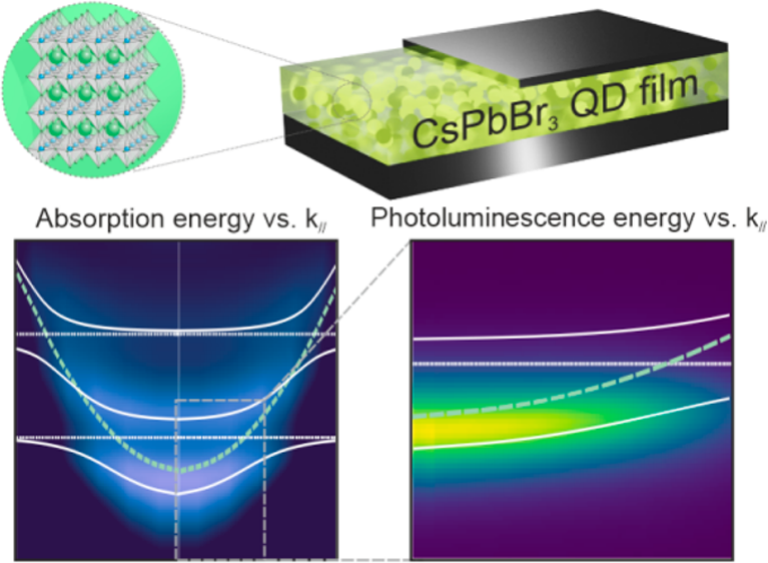

Strong coupling between lead halide perovskite materials and optical resonators enables both polaritonic control of the photophysical properties of these emerging semiconductors and the observation of fundamental physical phenomena. However, the difficulty in achieving optical-quality perovskite quantum dot (PQD) films showing well-defined excitonic transitions has prevented the study of strong light–matter coupling in these materials, central to the field of optoelectronics. Herein we demonstrate the formation at room temperature of multiple cavity exciton-polaritons in metallic resonators embedding highly transparent Cesium Lead Bromide quantum dot (CsPbBr_3_-QD) solids, revealed by a significant reconfiguration of the absorption and emission properties of the system. Our results indicate that the effects of biexciton interaction or large polaron formation, frequently invoked to explain the properties of PQDs, are seemingly absent or compensated by other more conspicuous effects in the CsPbBr_3_-QD optical cavity. We observe that strong coupling enables a significant reduction of the photoemission line width, as well as the ultrafast modulation of the optical absorption, controllable by means of the excitation fluence. We find that the interplay of the polariton states with the large dark state reservoir plays a decisive role in determining the dynamics of the emission and transient absorption properties of the hybridized light-quantum dot solid system. Our results should serve as the basis for future investigations of PQD solids as polaritonic materials.

## Introduction

Exciton-polaritons arise as a result of the strong coupling between confined photons and bound electron–hole pairs,^1^ and are thus characterized by their hybrid light–matter nature.^2−4^ This hybridization takes place within designed optical environments, such as optical cavities, in which the electromagnetic field intensity is magnified for specific photon energies selected to match those of the targeted electronic transitions.^5,6^ The exploration of this interaction in the field of lead halide perovskite materials has given rise to polaritonic controlled optical absorption and emission in film-shaped,^7,8^ microcrystalline,^9,10^ nanosized^11,12^ (namely, nanowires,^13^ nanoplatelets,^14,15^ and nanocubes^16^) and low-dimensional (such as Ruddlesden–Popper phases^17^) perovskites, which has been put into practice to develop optical switches,^18^ lasers,^13,15,19^ solar cells,^8^ light emitting diodes,^10^ sensors,^20^ and photodetectors^21^ with enhanced performance. Reciprocally, the integration of these emerging materials in the field of polariton physics has provided the opportunity to observe fundamental phenomena.^22−25^ However, the observation of strong light–matter coupling in PQD solids has remained elusive, in spite of being some of the most appealing materials for both fundamental analysis and applications in optoelectronics.^26−29^ Furthermore, this interaction has been scarcely investigated in QD solids in general, regardless of their composition, with only a few examples employing extremely thin CdSe and CdZnS/ZnS QD films.^30,31^ The reason for this is 3-fold. First, there is the difficulty to build QD films of high optical quality (i.e., scattering free), which hinders their integration in an optical cavity, thus avoiding well-defined optical resonances to be achieved. Second, the weak oscillator strengths of excitonic transitions in QD solids at room temperature, which results from the electronic energy disorder originating from the size dispersion of the as-synthesized QDs and, in the case of PQDs, also from the characteristic low exciton binding energy.^32^ Third, QDs may suffer from spectral diffusion,^33^ which causes fluctuations in the electronic energy transitions.^34^ In this context, recent advances to improve the quality of colloidal PQDs^35^ have permitted to attain transparent films made from cubic CsPbBr_3_ nanocrystals, which have been integrated into a photonic crystal resonator to observe weak light–matter coupling properties, as evidenced by the observation of amplified spontaneous emission.^36,37^ In another recent achievement, it has been shown that PQD films, whose absorption spectra partially preserve the excitonic features present in the colloidal cubic CsPbBr_3_ nanocrystals used as building blocks, exhibit the excitonic optical Stark effect under very intense photoexcitation.^38^ Both results imply substantial advancement in the field of perovskite photonics.

In this paper, we demonstrate a robust procedure to achieve strong light–matter coupling between a metallic optical cavity and a PQD solid. Central to this achievement are recent advancements in the preparation of CsPbBr_3_ QDs with ultrahigh monodispersity,^39^ which allows us to build uniform, large-scale, thick (∼500 nm), transparent PQD films capable of sustaining well-defined excitonic transitions. Following a rational design of the microcavity, hybrid light–matter states are formed, as could be unequivocally confirmed by the analysis of the absorption energy dispersion relation. From a practical perspective, the reconfiguration of the electronic and photon states gives rise to a significant reduction of the photoemission line width as well as provides the possibility to controllably tune the coupling strength, and thus Rabi splitting, by means of the excitation fluence, with a modulation time as fast as one ps and a recovery time of the order of a few nanoseconds. Furthermore, analysis of the excitation and relaxation dynamics of the PQD optical cavity indicates that they can be explained without considering the effects of biexciton interaction or large polaron formation, which have been pointed out as partially responsible for the reported ultrafast response of PQDs. Instead, we find that the interplay of the polariton states with the large dark state reservoir, an exclusive feature of hybridized light–matter systems, plays a decisive role in determining the dynamics of the transient absorption and emission properties. Overall, our results lay the groundwork for future investigations of PQD solids as polaritonic materials and their potential application in optoelectronics.

## Results and Discussion

**Figure 1:**
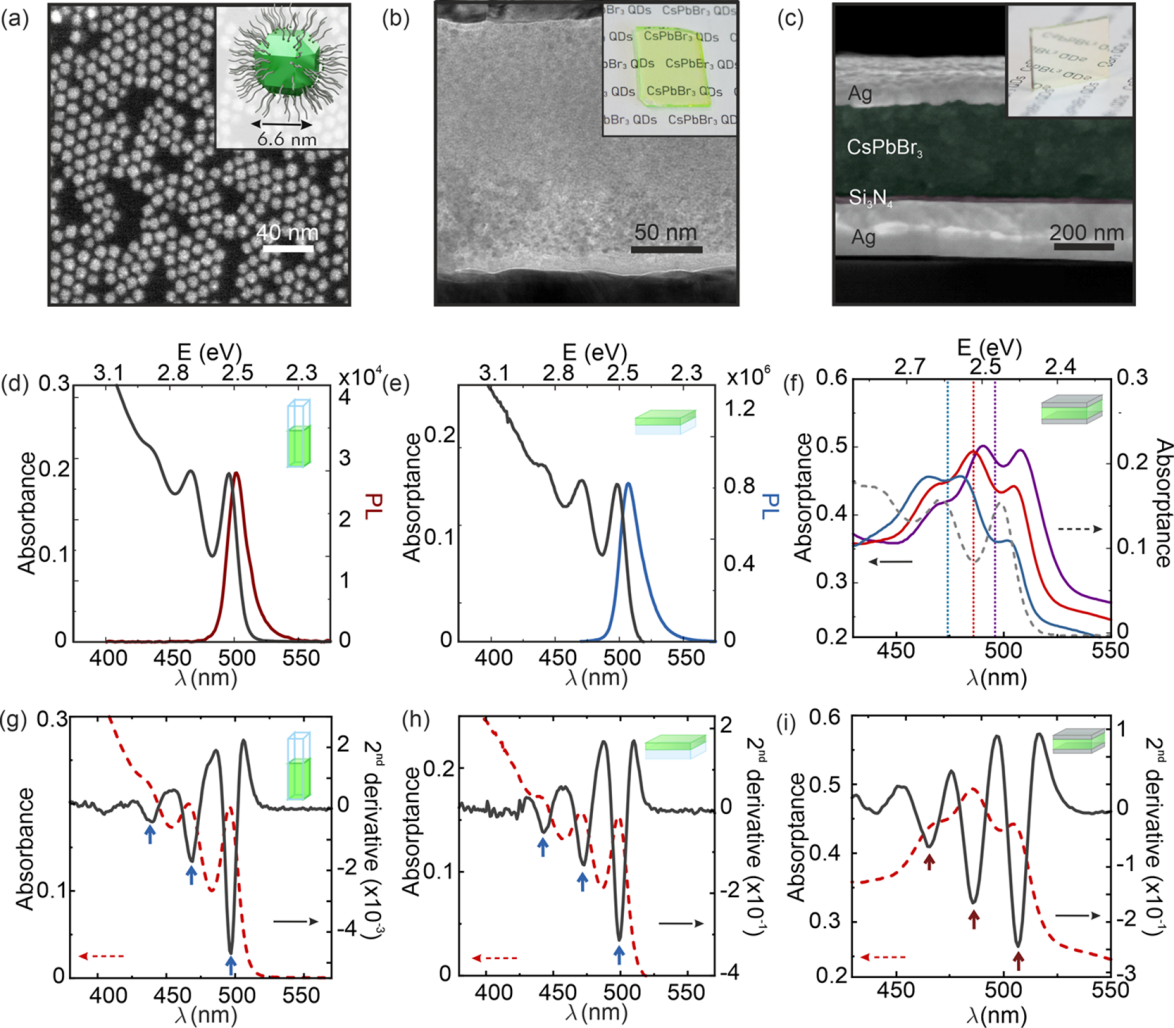
Electron microscopy and linear optical absorption. (a) TEM image of CsPbBr_3_-QDs dispersed on a grid; inset shows the schematic structure of a 6.6 nm lecithin-capped spheroidal CsPbBr_3_ nanocrystal. (b) HRTEM image of a spin-casted CsPbBr_3_-QD film cross section; inset shows a photograph of such film. (c) SEM image of a cross-section of a CsPbBr_3_-QD metallic optical cavity; inset shows a photograph such cavity. (d) Absorbance and photoluminescence (black and red curve, respectively) spectra of a 0.73 mg/mL CsPbBr_3_-QD dispersion in toluene. (e) Absorptance and photoluminescence (black and blue curve, respectively) spectra of a CsPbBr_3_-QD film. (f) Absorptance spectra of the PQD cavity at angles of incidence 26°, 36°, and 46° (purple, red and blue, respectively) attained with unpolarized light. The dotted colored lines represent the position of the reflectance minima of the third order optical cavity mode at each angle (same color code); the absorptance of the bare film at 0° (gray dashed line) is plotted for the sake of comparison. (g-i) Second derivative of the absorption spectra (black solid lines) compared with absorptance spectra (red dashed lines) of CsPbBr_3_-QDs (g) dispersion, (h) thin film, and (i) cavity. Blue and red arrows indicate the minima in the second derivatives that correspond to excitonic and polaritonic transitions, respectively.

CsPbBr_3_-QDs investigated in this work are capped with long-chain zwitterionic lecithin ligands and synthesized by the recently developed slow-growth room-temperature method, which yields QDs with a very high monodispersity and size-tunability.^39^ These CsPbBr_3_-QDs present a spheroidal rhombicuboctahedral shape with an average diameter of 6.6 nm (Figure 1a) and show two exceptionally well-resolved excitonic transitions in the absorbance spectrum (Figure 1d) when diluted with toluene (0.73 mg/mL). Concentrated solutions (88 mg/mL) were spin-coated to prepare solid films after adding polystyrene (PS, 15 wt % with respect to that of CsPbBr_3_-QDs), which improves the quality and stability of the film (a comparative analysis of the effect of PS is shown in Supplementary Figure S1). The cast film presents high uniformity and transparency, as shown in the HRTEM image and inset of Figure 1b, respectively. Essential for the purpose of our work, the absorptance spectra of the film, plotted in Figure 1e, retain all the well-resolved excitonic transitions present in the colloidal dispersion. From both the colloidal dispersion and the film, intense photoluminescence (PL) emission is observed, with a QY of 77% for the dispersed QDs and 32% for the QD solid (QY = 25% in the case of the CsPbBr_3_-QDs without the addition of PS, Figure S1). Then, optical resonators were built by sandwiching the CsPbBr_3_-QDs transparent solid film between two silver mirrors. The cross-sectional SEM image displayed in Figure 1c shows the layered structure of the optical cavity, which consists, sequentially from bottom to top, of a 200 nm thick silver mirror, a 9 nm sputtered silicon nitride protective layer, a 360 nm thick film of CsPbBr_3_-QDs, and a thermally evaporated 30 nm silver mirror. The cavity length was tuned by varying the thickness of the CsPbBr_3_-QD film so that the third order resonance of the optical cavity matches the first excitonic transition of the perovskite system (please see Methods Section 1 and Figure S2). A series of absorption spectra measured with unpolarized incident light impinging at 26°, 36°, and 46° with respect to the cavity surface normal are depicted in Figure 1f (purple, red, and blue solid lines respectively). The spectra observed indicate a substantial reconfiguration of the absorption properties, with three new absorption peaks whose spectral positions coincide neither with those of the excitonic transitions of the bare CsPbBr_3_-QDs film (gray dashed line), nor with the underlying cavity modes^40^ at the selected angles (vertical dashed lines). This can be further confirmed by comparing the absorptance second derivative spectra, plotted in Figure 1g–i for all three CsPbBr_3_-QDs dispersion, film and optical cavity, respectively. Both excitonic and polaritonic transitions are readily recognized as minima in the spectra and are highlighted by blue and red arrows, respectively. The changes observed in the absorption, along with the significant angular dependence observed, are consistent with the formation of new hybrid states arising from the interplay between cavity photons and CsPbBr_3_-QDs excitons.

**Figure 2:**
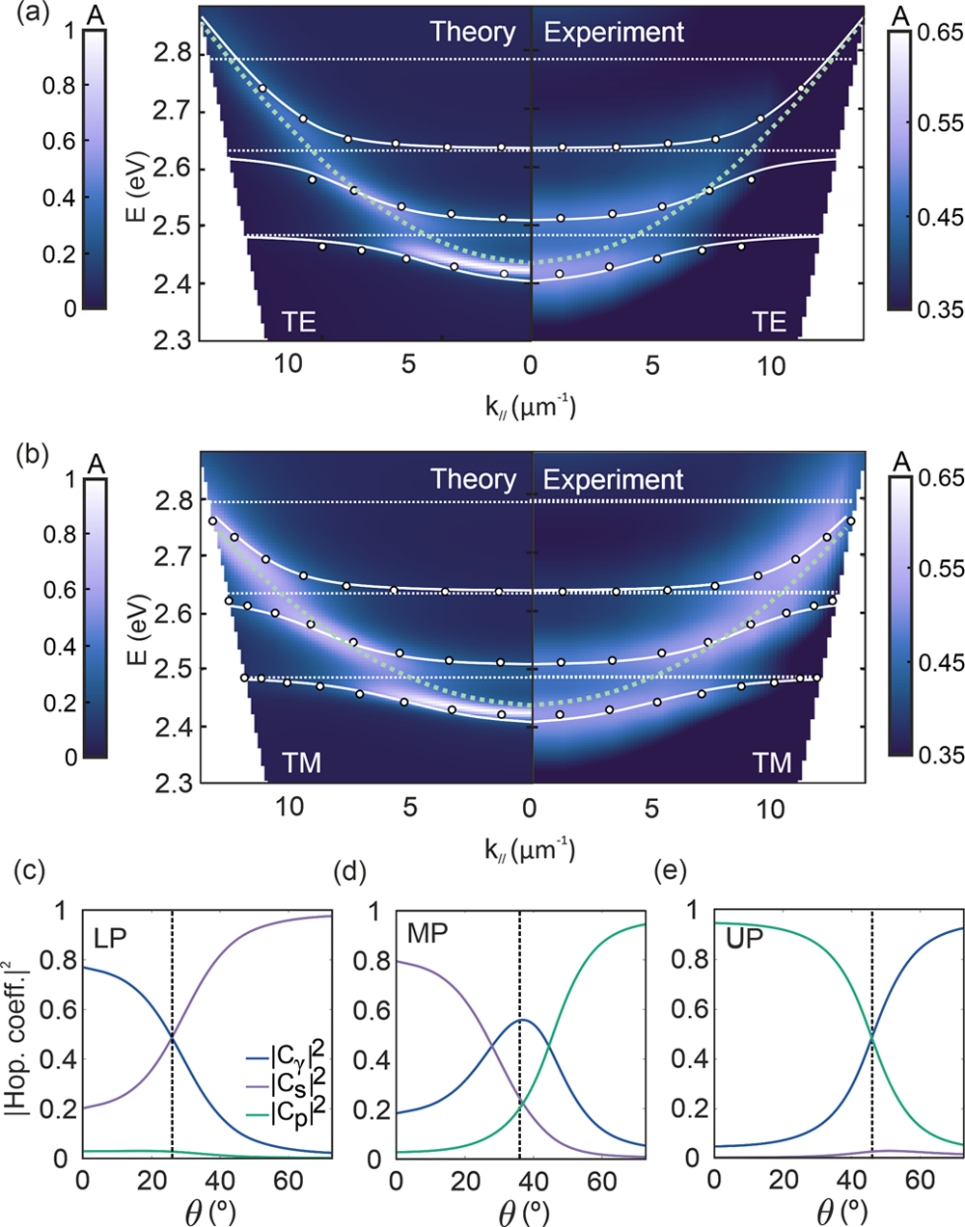
Exciton-polariton energy dispersion relation for a CsPbBr_3_-QD cavity. Theoretical (left panel) and experimental (right panel) absorptance energy dispersion maps for (a) TE and (b) TM polarizations of the incident beam. White dots indicate the spectral position of the experimental absorption maxima, horizontal dotted white lines indicate the positions of the first three excitonic transitions, green dotted lines represent the underlying cavity mode dispersion, and solid white lines are the LP, MP, and UP dispersions attained by solving the Tavis-Cummings Hamiltonian, assuming oscillator strengths *f*_s_ = 0.75 and *f*_p_ = 0.46 and N/V_C_ = 2.159 × 10^18^ QDs/cm^3^ (see Section 2 of the Supplementary Methods). (c–e) Angular dependence of the squared Hopfield coefficients (|*C*_γ_|^2^, |*C*_s_|^2^ and |*C*_p_|^2^, plotted as blue, purple, and green lines, respectively) attained from the three coupled oscillator model for the (c) LP, (d) MP, and (e) UP; vertical dashed lines indicate the angles at which the photon and exciton contribution to the coupling have a similar weight for each polariton.

Energy dispersion absorption maps are attained by plotting the absorptance versus the photon energy (*y*-axis) and the parallel component of the wavevector **k**_∥_ (*x*-axis, **k** = **k**_∥_ + **k**_⊥_) for the two polarizations of the incident beam studied, namely transversal electric (TE, Figure 2a) and transversal magnetic (TM, Figure 2b). For the sake of comparison, simulated maps (left panels in each figure) calculated using the transfer matrix method (please see Methods section) are plotted alongside the experimental ones (right panels), showing a fair agreement. The optical constants of the PQD solid used for the calculations were determined experimentally from the transparent films and are shown in Figure S3. The dispersion of the third order cavity mode is drawn as a green dotted line, while horizontal white dotted lines indicate the positions of the first (1s–1s, ℏω_s_), second (1p–1p, ℏω_p_) and third (1d–1d, ℏω_d_) excitonic transitions of the CsPbBr_3_-QD film.^39^ Analysis of the results shown in Figure 2 confirms that the strong coupling between exciton and photon modes leads to the formation of hybrid light–matter states in the ensemble. These are featured by three clearly distinguishable polaritonic branches (lower, middle, and upper polaritons: LP, MP and UP) and the opening of energy gaps resulting from the anticrossing of such branches. Energy gaps are estimated at the crossing point between the cavity dispersion curve and the exciton transition position, and, consistently, present a value of ℏΩ_R1_ = 87 ± 5 meV between LP and MP and of ℏΩ_R2_ = 77 ± 5 meV between the MP and UP, where ℏ is the reduced Planck constant and Ω_R_ is the Rabi frequency, which indicates the rate at which energy is exchanged between the photonic mode and the excitonic transitions. The magnitude of these gaps, also known as Rabi splittings, depends on the number of QDs effectively contributing to the coupling (*N*), the oscillator strength of the targeted transition (*f*) and the effective cavity volume occupied by the optical resonance involved in such coupling (*V*_C_) as
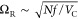
1In fact, cryogenic experiments demonstrate that ℏΩ_R1_ is gradually enlarged up to almost 30% due to the increase of *f* with decreasing temperature, caused by the reduction of phonon scattering, reaching ℏΩ_R1_ = 112 ± 5 meV at 80 K, as shown in Supplementary Figure S4. Further insight into the PQD solid-cavity coupling may be obtained by solving the Tavis-Cummings Hamiltonian, *Ĥ*_TC_, which describes a system of *N* noninteracting particles coupled to a single cavity light mode in the low excitation limit.^40,41^ Within this framework, we can obtain the theoretical polariton dispersions, plotted as white solid lines in Figure 2, which show very good agreement with those experimentally estimated, and the Hopfield coefficients, *C*_γ_, *C*_s_, and *C*_p_, whose squared values give us the degree of contribution to the coupling of the cavity mode and of each excitonic transition, respectively. These coefficients are plotted versus the angle of incidence of incoming light with respect to the cavity normal in Figures 2c–e. From these, it can be readily seen that LP and UP are mainly participated by the cavity mode and either the first (1s–1s) or the second (1p–1p) excitonic transition, respectively, while the MP shows a much more even contribution from all states. In Figure S5, we plot the calculated absorptance, as well as the spatial and spectral profiles of both the intensity of the electric field, |**E**(**r**)|^2^, and the corresponding absorbed luminous power, P_A_, along a cross-section of a CsPbBr_3_-QDs filled optical cavity for those angles for which the respective contribution of the optical mode and the excitonic transitions to the coupling is approximately 50% (highlighted as vertical dashed lines in Figures 2c-e). Full details on the solution of *Ĥ*_TC_ are provided in the Supplementary Methods, Section 2. It should be remarked that the vast majority of solutions, whose energies lie between those of the LP, MP, and LP, cannot be accessed from the ground state for symmetry reasons and are referred to as dark states. Interestingly, although this large reservoir of dark states is not observable in a linear absorption experiment, it significantly contributes to determining the dynamics of both the ultrafast transient absorption and PL decay properties of the PQD cavities, as will be shown further below. For the sake of comparison, and in order to illustrate the versatility of the method, along with the dependence of both the energy dispersion and the Hopfield coefficients on the cavity design, results for a cavity made of a 600 nm thick CsPbBr_3_-QD film are also shown in Figure S6.

**Figure 3:**
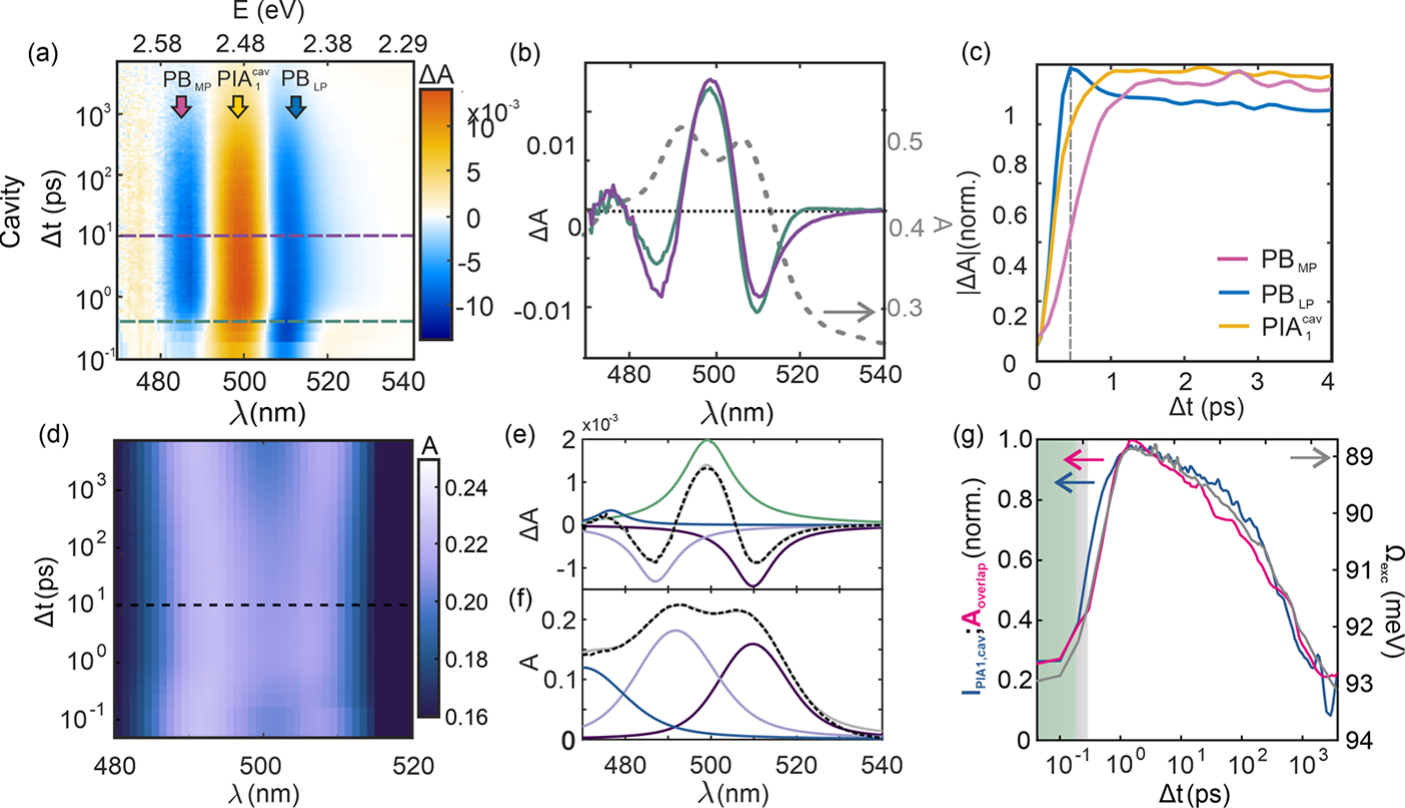
Ultrafast transient absorption spectroscopy of CsPbBr_3_-QD film and optical cavity and ultrafast modulation of the Rabi splitting. (a) Map of ΔA versus λ and Δ*t* for the CsPbBr_3_-QD optical cavity, in which the main signals are labeled. (b) Some illustrative selected Δ*A* spectra, attained at Δ*t* = 0.4 ps (green line) and Δ*t* = 10 ps (purple line); both Δ*t* are highlighted by dashed horizontal lines in (a). The corresponding linear absorptance spectra are also plotted (gray dashed lines). (c) Early time evolution of the maximum intensity of the main signals extracted from the analysis of |Δ*A*|. (d) Map of the reconstructed linear absorptance versus Δ*t* and λ. (e) Illustrative example of the breakdown of a selected Δ*A* spectrum (Δ*t* = 10 ps, dashed violet horizontal line in panel (a) into its main component signals: PB_LP_, PB_MP_ and PIA (dark violet, light violet and green solid lines, respectively); gray solid curve shows the sum of all three contributions. (f) Illustrative example of the breakdown of a selected reconstructed linear absorptance (Δ*t* = 10 ps, dashed horizontal line in panel (d)) into a LP, MP, and UP absorption peaks (dark violet, light violet and blue lines, respectively). Experimental curves and fittings are also plotted (black dashed and gray solid lines, respectively). (g) Comparison between the evolution of the maximum intensity of *I*_PIA_ and *A*_overlap_ (blue and pink solid lines, respectively, left *y*-axis) and that of Ω_exc_ (gray solid line, right *y*-axis) as extracted from the fittings shown in (e) and (f). The green and gray shaded regions in f highlight the duration of the excitation pulse, Δ*t* = 190 fs, and the estimated time for carrier thermalization, Δ*t* ≈ 350 fs, respectively.^42^

Polariton excitation and decay dynamics in CsPbBr_3_-QD optical microcavities were studied by ultrafast transient absorption spectroscopy (TAS). Results are presented as intensity maps in Figure 3a, in which the full series of Δ*A* spectra (where Δ*A* is the result of subtracting the absorbance of the nonexcited cavity from that of the photoexcited one) are plotted as a function of probe photon wavelength and pump–probe delay, Δ*t*, following the nonresonant excitation (i.e., not matching the polaritonic transitions in the cavity) at time 0 by a 190 fs, λ = 420 nm and ∼120 μJ/cm^2^ laser pulse. Under these conditions, we obtain *N*_e–h_ ≈ 0.6 excitations per QD, well below saturation, as demonstrated in Figure S7, preventing the damage of the samples. Selected Δ*A* spectra attained at different delay times, namely, at Δ*t* = 0.4 ps (green line) and Δ*t* = 10 ps (purple line), are explicitly shown in Figure 3b, while the early stage dynamics of the main signals identified in Figure 3a are plotted in Figures 3c. For the sake of comparison, a similar analysis, which is provided in the Supporting Information (Figure S8 and Supplementary Methods, Section 3) was performed for the CsPbBr_3_-QD colloidal dispersion and the bare film. The geometry of the experiment employed for each type of sample is described in the Methods Section.

Two prominent photobleaching signals are detected in Figure 3a at the lower (PB_LP_) and middle (PB_MP_) polariton spectral positions, which show a very different rise time (τ_PB,LP_ < 0.5 ps, τ_PB,MP_ < 2 ps) as can be seen in Figure 3c (blue and pink lines). In this regard, while the bleaching of the LP may be attributed to filling of the lowest energy states due to hot-electron relaxation, the subsequent long-lived bleaching of the MP cannot be understood without considering the interplay with the large reservoir of dark states. Even though not directly accessible from the ground state, dark states can be filled both from the lower polariton state and from higher energy levels as hot electrons cool down.^43^ This picture is further supported by the partial recovery of the absorption evidenced by the peak observed in the PB_LP_ signal at Δ*t* = 0.45 ps (signposted by a vertical gray dashed line in Figure 3c), which points at a transfer of carriers from the lower polariton state to the dark state reservoir once a certain occupation level is attained. Thus, gradual filling of the dark states could eventually lead to the transfer of carriers to the middle polariton state, giving rise to its bleaching. These carrier exchanges are favored by the significant overlap between dark and polaritonic states,^44,45^ which can be inferred from the moderate separation between lower and middle polaritons we observe.

As for the intense PIA signal observed between PB_LP_ and PB_MP_, a detailed analysis of the TAS signal indicates that it is the result of the photoinduced reduction of the polariton absorption splitting. In fact, nonresonant photopumping could give rise to the inhibition of the Rabi splitting, as a consequence of either the partial saturation of available excitonic states or the screening of the exciton transition dipole moment by electron–hole pairs supported by the interconnected QD network, as expressed in the formula^46^
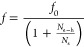
2, in which *f*_0_ is the oscillator strength in the absence of pumping, and *N*_*e-h*_ and *N*_*s*_ are the pump generated and saturation densities, respectively. Considering the relation between *f* and the Ω_R_ given by eq 1, the absorption splitting of a polaritonic system under intense photoexcitation (Ω_exc_) is reduced, from its linear absorption value Ω_0_, to
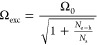
3The narrowing of Ω_exc_ becomes explicit when we plot the result of modulating the linear absorptance with the ΔA data attained from TAS at all Δ*t*, as shown in Figure 3d. In this representation, the intense PIA signal observed may be understood as the result of the smaller energy separation between LP and MP absorption peaks, which gives rise to newly available states in the spectral region in which there was a gap initially, thus allowing transitions from the ground state that were not present in the pump-off state. For the UP a similar PB_UP_ is observed, as well as, accordingly, the corresponding PIA between MP and UP (Figure S9). To support this hypothesis, from the fitting of the TAS signal at all *Δt* (an example is shown in Figure 3e for Δ*t* = 10 ps) and that of the reconstructed absorptance spectra (as exemplified in Figure 3f, also at Δ*t* = 10 ps), we extract the time evolution of both the PIA signal and Ω_exc_, estimated as the spectral difference between the maxima of the two peaks corresponding to the transitions from the ground state to the lower and middle polaritons (*A*_LP_ and *A*_MP_). The comparison of the PIA signal intensity, *I*_PIA_, and Ω_exc_ versus Δ*t* is shown in Figure 3g (gray and blue lines, respectively). The time dependence of the overlapping area of A_LP_ and A_MP_, A_overlap_, is also plotted (pink solid line, Figure 3g). This plot reveals a strong correlation between the splitting reduction and the occurrence of the observed PIA, hence, further supporting their causal link. Also, from this analysis and eq 3, we can estimate a saturation e^–^–h^+^ density per QD of Ns ≈ 3.5, considering that Ω_0_ ≈ 93 meV and that Ω_exc_ ≈ 89 meV for an e^–^–h^+^ density N_e–h_ ≈ 0.6.

It should be remarked that a similarly pronounced PIA, spectrally located between the 1s–1s and 1p–1p excitonic transitions, has been reported for CsPbBr_3_-QDs dispersions,^47,48^ and it is also observed in our bare films (Figure S8). Its origin has been attributed to either the relaxation of selection rules due to the formation of large polarons,^49,50^ which might enable parity forbidden exciton transitions,^47^ or to higher energy exciton-biexciton transitions.^48^ Interestingly, none of these phenomena seem to be playing a significant role in the ultrafast response of the photon-dressed excitons formed in the cavity. From a more applied perspective, these results demonstrate the possibility to attain ultrafast modulation of the exciton-polariton absorption in cavity coupled QD solids, an effect that has also been shown for molecular materials,^51^ semiconductor microcavities,^52^ and transition metal dichalcogenides^53^ coupled to surface plasmons. In those cases, the duration of the Rabi splitting reduction reported was 3 orders of magnitude shorter than in our case, in which the linear absorptance is fully recovered (thus the PIA disappears) at Δ*t* ≈ 10 ns, when all the photoexcited e^–^–h^+^ pairs decay back to the ground state.

**Figure 4:**
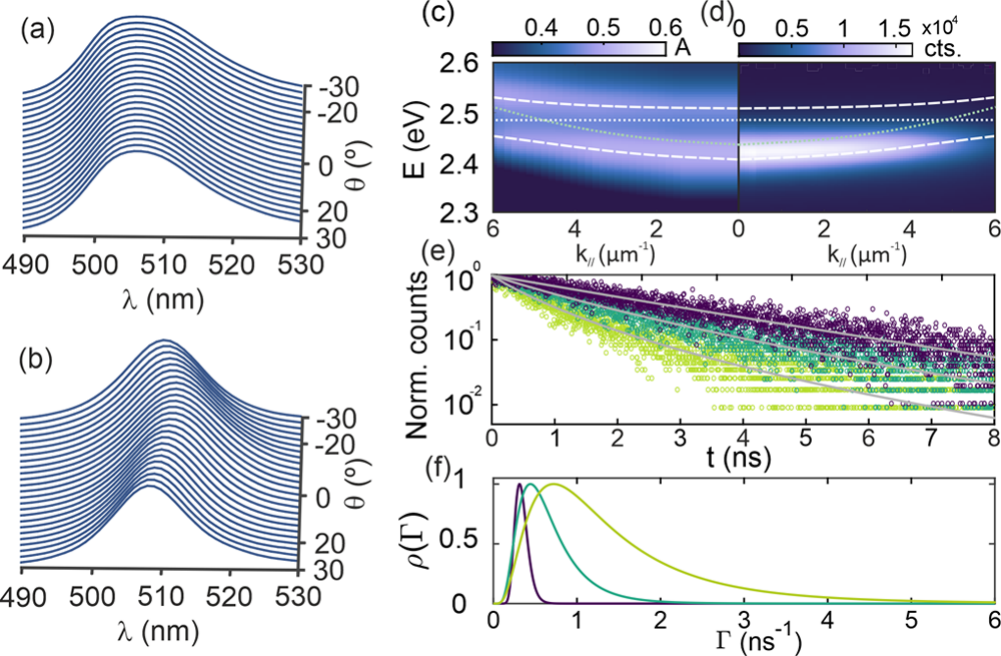
Static and dynamic photoemission properties of CsPbBr_3_-QD dispersion, film and optical cavity. Normalized PL spectra at different collection angles (from −30° to 30°) of the (a) bare film and (b) optical cavity. Comparison of the optical cavity (c) absorptance and (d) PL dispersions along *k*_*||*_. The white dotted horizontal line indicates the spectral position of the original excitonic transition, the light green dotted curve shows the mode underlaying cavity mode dispersion, and the white dashed lines are the theoretical results attained by solving the Tavis-Cummings Hamiltonian. (e) PL decay curves attained for the CsPbBr_3_-QD colloidal dispersion, film, and optical cavity (violet, dark green, and light green open circles, respectively) together with their corresponding fittings according to a log-normal distribution of decay rates (gray solid lines). For the sake of comparison, also in this case, data were obtained for a similar number of photoexcitations per CsPbBr_3_-QD (*N*_exc_ ≈ 0.016). (f) Decay rate distributions, ρ(Γ), attained for the best fittings of the PL decay curves for the CsPbBr_3_-QDs dispersion, film, and optical cavity (violet, dark green, and light green solid lines, respectively).

The photoemission of the strongly coupled PQD solid-cavity system also undergoes significant spectral and directional reconfiguration. In Figure 4, the PL spectra measured from either a CsPbBr_3_-QD film on quartz (Figure 4a) or from a strongly coupled CsPbBr_3_-QD cavity (Figure 4b) are shown. Measurements were taken using a back focal plane spectroscopy set up, which allows us to collect the emitted radiation in a wide angular range (−30°<θ < 30°) by scanning the Fourier plane with an optical fiber coupled to a CCD detector, i.e., without tilting the sample and hence assuring that all spectra are collected from the same spot and under the same photoexcitation intensity from a continuum diode laser emitting at λ = 450 nm. Analysis of these measurements shows that the CsPbBr_3_-QD cavity is determined by the spectral dispersion and the line width of the lower polariton transition^54^ as can be explicitly seen in the comparison between the polariton absorption and emission made in Figure 4c. In this case, absorptance was estimated also using back focal plane spectroscopy. The horizontal dashed line indicates the spectral position of the first uncoupled excitonic transition, while the white solid line curves show the angular dispersion of the LP and MP. A very significant 35% reduction of the spectral photoemission line width, from 96 meV in the film to 62 meV in the cavity, is observed. Emission occurring from middle and upper polaritons was not detected, since higher energy polariton states have a very short lifetime due to nonradiative decay to the large number of incoherent states present in the dark state reservoir.^55^

The dynamic properties of the excited states were studied by probing the time evolution of the emission with a time-correlated single photon counting (TCSPC) setup. The same ultrafast pulsed laser employed for TAS measurements emitting at λ = 420 nm was employed to photoexcite the samples, whose emission was collected with an avalanche photodiode. In Figure 4e we show PL decay curves attained for all three systems under study, together with their corresponding fits assuming a log-normal distribution of decay rates (gray solid lines, see Suplementary Methods, Section 6, for details). For the sake of comparison, we have plotted a set of data obtained for a similar number of photoexcitations per CsPbBr_3_-QD (*N*_exc_ ≈ 0.016, be it in suspension, in the film, or as part of the optical cavity). The full collection of PL decay curves measured for different fluences for each sample can be consulted in Supplementary Figure S10. Analysis of the decay rate distributions, ρ(Γ), which are drawn in Figure 4f, reveals that the CsPbBr_3_-QD film presents a much wider range of decay paths with respect to the colloid, as the much narrower ρ(Γ) of the latter indicates. Accordingly, the most likely lifetime (τ, corresponding to the Γ for which ρ(Γ) is maximum, Γ_max_) is also significantly higher in the CsPbBr_3_-QDs dispersion (τ = 3.23 ns) than in the film (τ = 2.22 ns), which implies that not only the variety but also the density of nonradiative states increases in the film. The presence of a higher density of nonradiative pathways in the film is likely the consequence of the formation of multiple interfaces due to the CsPbBr_3_-QDs packing. In turn, the polaritonic system presents an even broader ρ(Γ) and shorter characteristic τ (τ = 1.35 ns) than the film, which agrees with the presence of a large reservoir of nonradiative dark states to which photoexcited carriers can be transferred from the LP level. These results are therefore also in line with those attained from the analysis of the TAS signal, which also supports the idea that relaxation dynamics strongly depend on the interplay with the dark states.

## Conclusions

In conclusion, we demonstrated strong light–matter coupling between a CsPbBr_3_-QD solid film and an optical cavity. Central to this achievement is the preparation of scattering-free films made of highly monodisperse CsPbBr_3_ nanocrystals, displaying up to three well-resolved excitonic transitions in the absorption spectra. This allows us to fabricate metallic optical resonators with enough quality as to give rise to the formation of three polaritonic branches (namely upper, middle, and lower), as a result of the superposition of two excitonic and one optical cavity excitations, as could be unequivocally confirmed by analysis of the absorption energy dispersion relation. Comparative analysis of both the ultrafast transient absorption and the photoluminescence decay of all three CsPbBr_3_-QDs systems under analysis (i.e., dispersion, film and optical cavity), reveals a very different dynamics in the case of photon-dressed excitons in the resonator with respect to bare ones in the CsPbBr_3_ nanocrystals. Our results indicate that the effects of biexciton interaction or large polaron formation, frequently invoked to explain the transient absorption properties of PQDs, are seemingly absent or compensated by other more conspicuous effects in the CsPbBr_3_-QDs optical cavity. If the inhibition of polaron formation is confirmed, this would imply that charge transport in cavity-coupled QD solids should differ from that in standard perovskite materials, as photoinduced lattice distortions have been suggested to play a key role in their conductivity.^49^ Instead, we find that the interplay of the polariton states with the large dark state reservoir plays a decisive role in determining the dynamics of the transient absorption and emission properties of the hybridized light–matter system, which must be fully explored to harness the full potential polaritonics bear for optoelectronics and information technology. From a practical perspective, the reconfiguration of the electronic and photon states gives rise to a significant reduction of the photoemission line width as well as provides the possibility to controllably tune the coupling strength, and thus Rabi splitting, by means of the excitation fluence, with a modulation time as fast as one picosecond and a recovery time of the order of a few nanoseconds. Our results should serve, overall, as the basis for future investigations of PQD solids as polaritonic materials and, in particular, as a necessary step toward the observation of Bose–Einstein condensation, which will allow leveraging the full potential of photon-dressed QDs in optoelectronics.

## Experimental Methods

### Colloidal CsPbBr_3_ Quantum Dot Synthesis

Lead bromide (PbBr_2_, 99.999%), cesium carbonate (Cs_2_CO_3_, 99.9%), hexane (≥99%), diisooctylphosphinic acid (DOPA, 90%), and acetone (ACE ≥ 99.5%) were purchased from Sigma-Aldrich. *n*-Octane (min. 99%) and lecithin (>97% from soy) were purchased from Carl Roth. Trioctylphosphine oxide (TOPO, > 90%) was purchased from Strem Chemicals. All chemicals were used as received.

PbBr_2_-TOPO stock solution (0.04 M) was prepared by mixing 4 mmol of PbBr_2_ with 20 mmol of TOPO in 20 mL of *n*-octane at 120 °C. The resulting solution was later diluted with 80 mL of hexane. Similarly, the Cs-DOPA solution (0.02 M) was prepared by mixing 100 mg of Cs_2_CO_3_ with 1 mL of DOPA in 2 mL of *n*-octane at 1200C and subsequently diluted in 27 mL of hexane. A 0.13 M lecithin stock solution was prepared by dissolving 1.0 g of lecithin in 20 mL of hexane. All stock solutions were filtered through 0.2 μL of PTFE before the use.

For the synthesis of 6.6 nm-large CsPbBr_3_-QDs, 30 mL of PbBr_2_-TOPO stock solutions were diluted with 180 mL of hexane, followed by the injection of 15 mL of Cs-DOPA stock solution under vigorous stirring. After 4 min of growth, 15 mL of lecithin stock solution were added. After a minute, the obtained solution was concentrated to 15 mL on a rotary evaporator and a 3-times volume excess of acetone acting as antisolvent was added. QDs were isolated by centrifuging at 20133 g for 1 min and redispersed in 24 mL toluene.

An additional washing with a toluene/ethanol pair of solvent/antisolvent was performed in order to remove the excess of lecithin. QDs were precipitated from the crude solution by adding 24 mL of ethanol and centrifuging the mixture at 20 133 g for 1 min. The product was redissolved in 12 mL of toluene, and washing was repeated with 12 mL of ethanol, followed by redissolution in 6 mL of toluene. On the third washing cycle, QDs were precipitated by 6 mL and, after centrifugation, redissolved in 2 mL of toluene. The obtained solution contained 88 mg/mL of QDs.

### CsPbBr_3_ QD Film and Cavity Preparation

Chemical reagents and solvents (i.e., polystyrene (PS) and toluene) were purchased from Sigma–Aldrich (highest grade available) and were used without further purification. A 2 wt % solution of PS in toluene was stirred at room temperature until complete dissolution of the polymer; then, the appropriate amount was added to the 88 mg/mL QDs dispersion in toluene in order to obtain a 15 wt % of PS with respect to the QDs. The resulting QDs/PS mixture in toluene was then spin-coated on the substrate by varying the spin-coating speed in order to adequately tune the cavity thickness. In order to prepare the cavity, substrates with 200 nm of silver and 9 nm of sputtered silicon nitrate were purchased from Fraunhofer and cleaned by ultrasonic bath (with 2% Hellmanex, acetone, and 2-propanol) followed by 10 min treatment with oxygen plasma. Then, on top of the CsPbBr_3_-QDs spin-coated layer, a silver mirror of 30 nm was thermally evaporated in vacuum at 10^–6^ mbar (Univex 250, Leybold vacuum), at 1 Å/s until reaching the desired thickness, by monitoring it with a quartz balance coupled to the system.

### Linear Absorption Characterization and Optical Constants Determination

Polarization and angular dependent measurements were conducted with double goniometer configuration (Universal Measurement Accessory, UMA), which allows us to rotate independently sample and detector and hence select arbitrary incident and collection directions, both attached to a UV–vis–NIR spectrophotometer (Cary 5000, Agilent). Absorptance (*A*) was attained as *A* = 1 – *R* – *T*, where *R* and *T* stand for reflectance and transmittance, respectively. The optical constants for the QD solid film were estimated by the method developed by Forouhi and Bloomer,^56^ based on the fitting of the experimental *T* and *R* measured at different angles of incidence and polarizations with the UMA. With this approach, we could estimate the spectral dependence of both real and imaginary components of the refractive index for the bare PQD solid film (Figure S3), which were then used to design the geometry of the optical cavity.

### Static and Dynamic Photoluminescence Analysis

Static photoluminescence was measured by back focal plane spectroscopy using a Leica DMI300 M microscope with a 100x, 0.75NA objective. The image from the sample’s back focal plane was directed to an optical fiber mounted on a motorized stage scanning the horizontal axis, each position of the fiber corresponding to a given angle of detection. The source of excitation is a 450 nm continuous laser, and the signal is detected by a UV–vis spectrophotometer (Ocean Optics. USB200).

Lifetime measurements were carried out with a TCSPC setup using an Ytterbium-doped potassium gadolinium tungstate (Yb:KGW, λ = 1040 nm) femtosecond pulsed laser (PHAROS PH1 from Light Conversion) operating at 1 kHz (pulse duration, 190 fs) sending 420 nm pulses and collecting the time-resolved photoluminescence with a single photon avalanche photodiode from MPD. The signal was processed employing commercial software provided by Ultrafast Systems.

### Ultrafast Spectroscopy Analysis

Ultrafast transient absorption spectroscopy (TAS) measurements were performed by using a pump-and-probe setup. The signal generated by a Yb:KGW pulsed laser is split into two beams. One is directed to an optical parametric amplifier (Orpheus, Light Conversion) where 420 nm pump pulses with a narrow spectral width (4 nm) are generated. The other beam passes through a delay line and, subsequently, a sapphire crystal to generate broadband probe pulses. Signal was collected with a CMOS detector attached to a spectrometer (HELIOS, Ultrafast Systems) in a transmission configuration for transparent samples (i.e., nanocrystal dispersions and thin films on quartz) and in a reflection configuration (with an angle of incidence and collection of the probe beam of 26) for opaque samples (metallic optical cavity). Collected signal is the result of TAS data are initially processed with Surface Xplorer software (background and chirp corrections) and further analysis (Voigt fit of transient absorption spectra) is performed with a Matlab code (See Supplementary Methods, Section 4, ). A constant scattering background was removed from the measurements before the fittings. The HELIOS Fire software produces a 3D wavelength-delay time-ΔA matrix, where ΔA is the result of subtracting the absorbance of the nonexcited sample from that of the photoexcited one, thus representing a differential absorbance . The density of e^–^–h^+^ per unit volume generated in the samples with the pump pulses are calculated, after correcting by the reflection, assuming that the excitation intensity over the volume of the sample is the average between the maximum intensity and the intensity attenuated after the absorption over one length of the sample (See Supplementary Methods, Section 5).^57^

### Optical Simulations

Optical reflectance, transmittance, and absorptance, as well as electric field intensity and absorption profiles, are calculated with a Matlab code using a transfer matrix method based on the Abeles formalism.^58^ The thickness of each layer is determined experimentally using a profilometer and the refractive indices are either taken from bibliography (Ag, Si_3_N_4_) or modeled through the fitting of the experimental *R* and *T* spectra using a Forouhi-Bloomer model of the dielectric constant (CsPbbr_3_-QDs layer).^56^ The spatial distribution of the EM field intensity along the direction of propagation at 0° (*z*), calculated in small intervals, is used to obtain the absorption per unit volume. This value, integrated in *z* between the limits imposed by the interfaces between layers, yields the theoretical absorptance of each layer in the ensemble.

## Data Availability

The data underlying this study are openly available in the Digital CSIC repository. The codes used for generating absorptance spectra and the spatial distribution of the optical field intensity and the absorption in an optical cavity are provided at https://github.com/Multifunctional-Optical-Materials-Group.

## Supporting Information Available

Effect of polysterene on CsPbBr_3_-QD film absorptance; refractive index and reflectance of the underlying cavity; optical constants of a CsPbBr_3_-QD film; absorptance and photoluminescence spectra at low temperature; visualization of optical mode splitting in cavity coupled CsPbBr_3_-QDs solids; polariton dispersion relation and Hopfield coefficients for a thicker CsPbBr_3_-QD solid coupled to an optical cavity; analysis of ultrafast TAS signal versus density of carriers; comparative analysis of ultrafast TAS of CsPbBr_3_-QD dispersion, film and optical cavity; upper polariton bleaching and higher order photoinduced absorptions; log-normal fit of PL decay curves; PL decay at different fluencies; Calculated dispersion relations attained considering different oscillator strengths; Tavis-Cummings Hamiltonian solutions: polaritons, dark states, and Hopfield coefficients (PDF)
