# Supplementary material for: Strong Light–Matter Coupling in Lead Halide Perovskite Quantum Dot Solids
Source: ACS Nano. 2024 Feb 1;18(6):4922–31. doi: 10.1021/acsnano.3c10358 (PMC10867889; doi:10.1021/acsnano.3c10358)
Supplement: Supplementary file 1 — nn3c10358_si_001.pdf [file nn3c10358_si_001.pdf]

Supplementary Information

Strong light-matter coupling in lead halide perovskite quantum dot solids

Clara Bujalance,[1,§] Laura Caliò,[1,§] Dmitry N. Dirin,[2] David O. Tiede,[1] Juan F. Galisteo-López,[1] Johannes Feist,[3] Francisco J. García-Vidal,[3] Maksym V. Kovalenko,[2] Hernán Míguez[1,]*

1 Multifunctional Optical Materials Group, Institute of Materials Science of Sevilla, Consejo Superior de Investigaciones Científicas – Universidad de Sevilla (CSIC-US), Américo Vespucio 49, 41092, Sevilla, Spain.

2 Laboratory of Inorganic Chemistry, Department of Chemistry and Applied Biosciences, ETH Zürich, CH-8093 Zürich, Switzerland; Empa – Swiss Federal Laboratories for Materials Science and Technology, CH-8600 Dübendorf, Switzerland.

3 Departamento de Física Teórica de la Materia Condensada and Condensed Matter Physics Center (IFIMAC), Universidad Autónoma de Madrid, 28049 Madrid, Spain.

Table of Contents

Supplementary Figures

S1. Comparative analysis of the effect of 15 wt% of polystyrene on $CsPbBr_3$-QDs film absorptance.

S2. Calculated Reflectance of the underlying cavity.

S3. Optical constants of a $CsPbBr_3$-QDs film.

S4. Absorptance and photoluminescence spectra at low temperature.

S5. Visualization of optical mode splitting in cavity coupled $CsPbBr_3$-QDs solids.

S6. Absorption energy dispersion relation and Hopfield coefficients for a 600 nm thick $CsPbBr_3$-QD solid coupled to an optical cavity.

S7. Analysis of ultrafast transient absorption spectroscopy signal versus density of carriers.

S8. Comparative analysis of ultrafast transient absorption spectroscopy results of CsPbBr3-QD dispersions, films and optical cavities.

S9. Complementary ultrafast transient absorption spectroscopy maps and dynamics: upper polariton bleaching and higher order photoinduced absorptions.

S10. Photoluminescence decay at different fluencies.

S11. Calculated dispersion relations attained considering different oscillator strengths.

Supplementary Methods

1. Refractive index and reflectance of the underlying cavity.
2. Tavis-Cummings Hamiltonian solutions: polaritons, dark states and Hopfield coefficients.
3. Comparative analysis of transient absorption spectroscopy results for PQD colloidal dispersions, thin films and optical cavities.
4. Transient absorption data treatment.
5. Charge carrier density estimation in TAS measurements.
6. Log-normal fit of PL decay curves.

Supplementary Figures

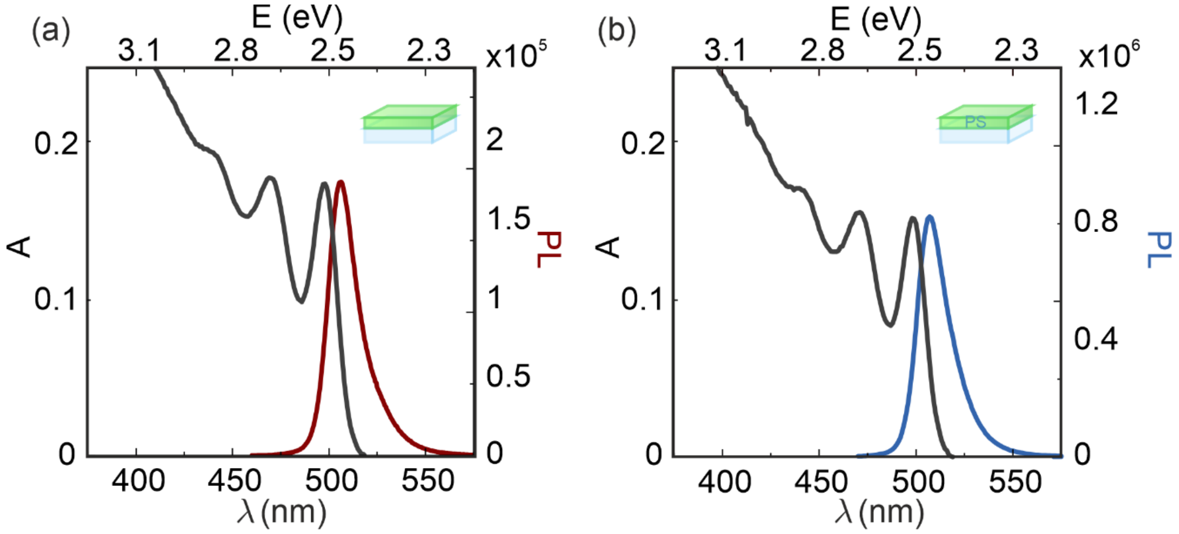

Figure S1. Absorptance(black) and photoluminescence (red/blue) spectra of the $CsPbBr_3$ film without (a) and with (b) polystyrene.

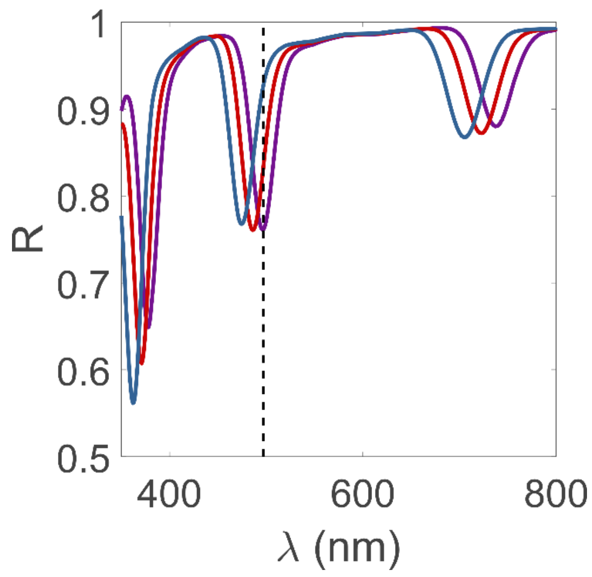

Figure S2. Simulated reflectance spectra of the assumed underlaying cavity at 26°, 36° and 46°(purple, red and blue, respectively), calculated using a constant index of 1.84. Only second, third and fourth order resonances are shown. The third order resonance coincides with the first excitonic transition of the $CsPbBr_3$-QD film.

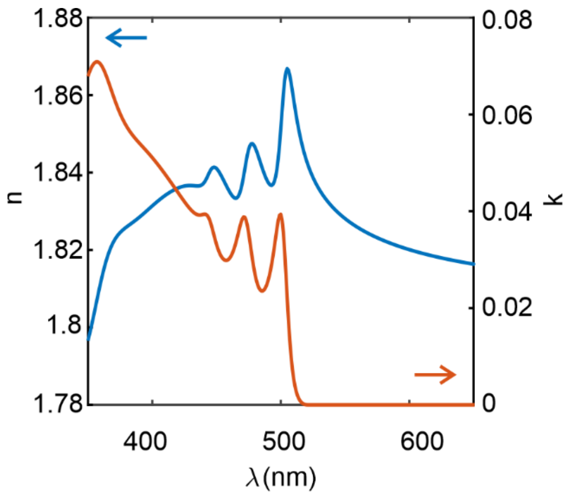

Figure S3. Real (n) and imaginary (k) parts of the refractive index of a film of $CsPbBr_3$ QDs with PS in blue and orange respectively.

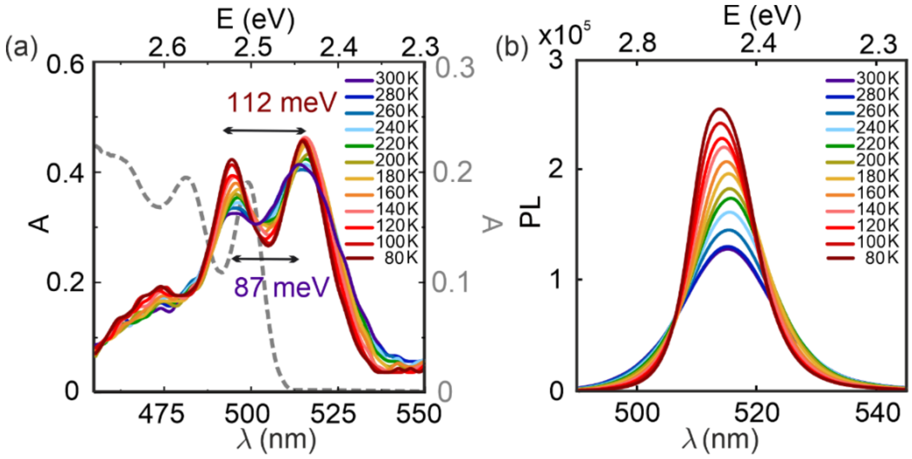

Figure S4. Absorptance (a) and photoluminescence (b) spectra of the $CsPbBr_3$ QDs + PS cavity measured from 300K to 80K.

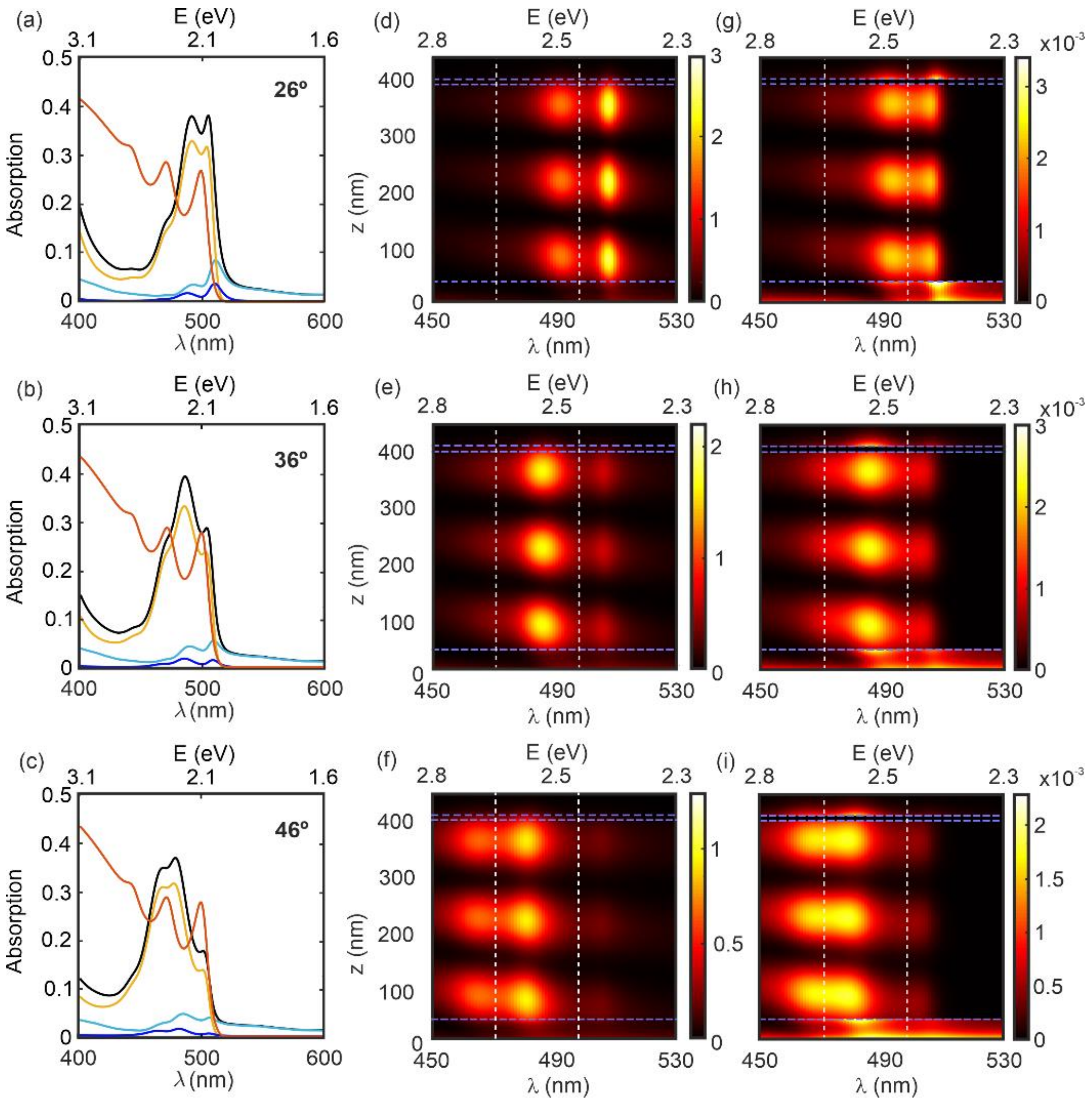

Figure S5. **Visualization of optical mode splitting in $CsPbBr_3$-QDs solids**. (a), (b) and (c), transfer matrix calculated spectra of the absorptance occurring at each layer in the ensemble, namely, top Ag (light blue line), $CsPbBr_3$-QDs (yellow line), bottom Ag (dark blue line) for 26°, 36° and 46° incidence angles, respectively. Black and orange lines are the absorptance of the whole cavity and a bare $CsPbBr_3$-QDs film with the same thickness at the same angles of incidence. (d), (e) and (f), calculated spatial and spectral profiles of the electric field intensity $|\mathbf{E}(\mathbf{r})|^2$, and, (g), (h) and (i), the absorbed luminous power P_A for the $CsPbBr_3$ cavity, for incidence angles of 26°, 36° and 46°. Calculations are performed considering a plane wave impinging on the top silver mirror (position 0 in the z-axis) and propagating along the z-direction. Interfaces between layers are indicated by horizontal dashed blue lines, while vertical white dashed lines represent the position of the 1s-1s and 1p-1p excitons. In this representation, both the order of the optical mode participating in the coupling and its splitting are evident by the number of nodes observed in the spatial profiles of both $|\mathbf{E}(\mathbf{r})|^2$ and P_A.

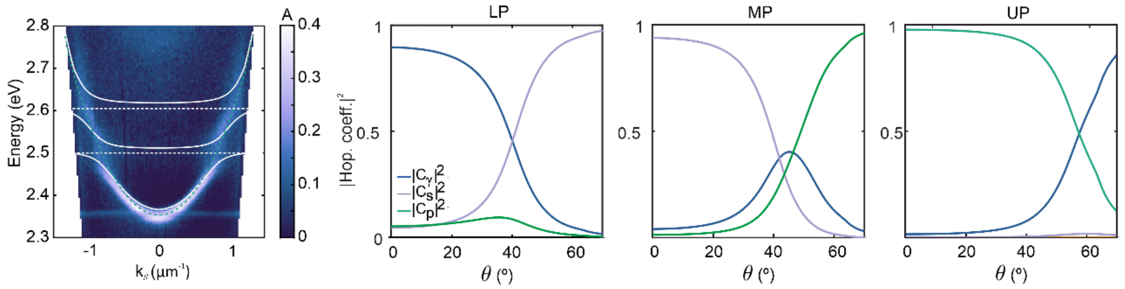

Figure S6. Absorption energy dispersion relation (left panel) and angular dependence of the corresponding Hopfield coefficients calculated for the lower, middle and upper polaritons (right panels) for a 600 nm thick $CsPbBr_3$-QD film embedded in a metallic optical cavity.

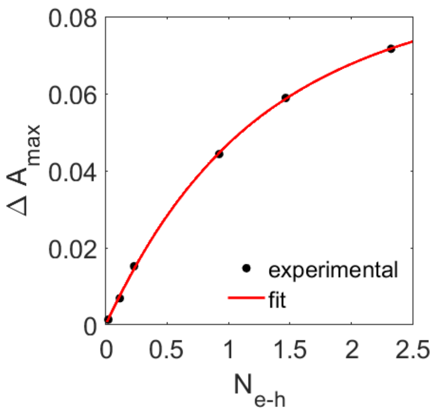

Figure S7. **Analysis of ultrafast transient absorption spectroscopy signal versus density of carriers.** Maximum bleach signal ΔA_{max} of the first exciton transition in the QD film versus the estimated $N_{e\text{-}h}$ (black scatters) and the fit to the phenomenological equation:[1] $\Delta A_{max} = \frac{\delta N_{e-h}}{\beta + N_{e-h}^{\gamma}}$

, where β, δ, and γ are fitting parameters. We obtain a characteristic saturation curve that proves that the relation between the estimated densities and the measured TAS signal follows the expected trend. It also shows that we are working far from saturation at the fluences employed in our TAS experiments ($N_{e\text{-}h}$=0.6 for the QD cavity TAS results shown in the main body of the manuscript).

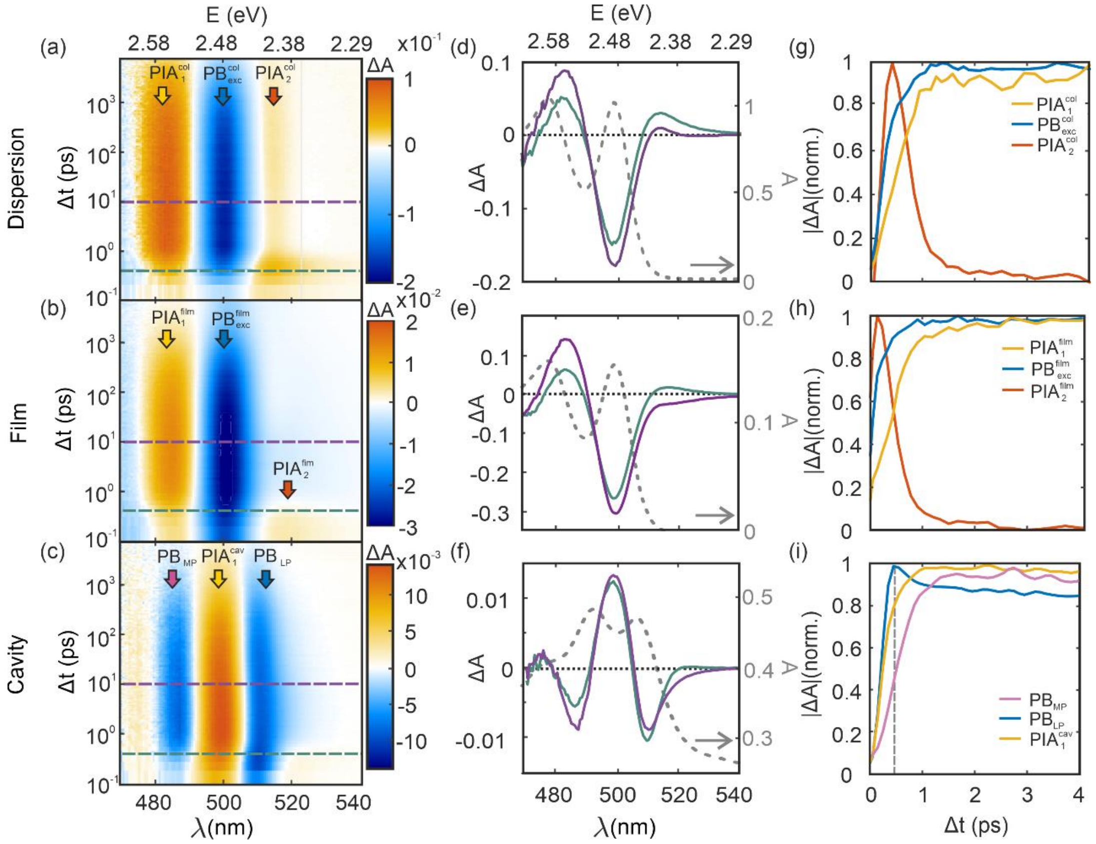

Figure S8. **Ultrafast transient absorption spectroscopy of $CsPbBr_3$-QDs dispersion, film and optical cavity**. Maps of ΔA versus λ and Δt for the $CsPbBr_3$-QD (a) dispersion, (b) bare film and (c) cavity, in which the main signals are labelled. Some illustrative selected ΔA spectra, attained at Δt=0.4 ps (green lines) and Δt=10ps (purple lines), are shown in (d), (e) and (f), respectively. Both Δt are highlighted by dashed horizontal lines in panels (a), (b) and (c). The corresponding linear absorptance spectra are also plotted (grey dashed lines). Panels (g), (h) and (i) display the early-time evolution of the maximum intensity of the main signals extracted from the analysis of |ΔA|. Pump wavelength and duration used for these experiments were λ=420 nm and 190 fs; fluences were varied in the range 12-118 $\mu J/cm^2$, depending on the sample absorptance at λ=420 nm, to ensure we have less than one excitation per PQD (please see section 3 in these Supplementary Methods,). Vertical grey dashed line in (i) indicates de maximum intensity of the LP photobleaching signal, which occurs at 0.45 ps. All cavity measurements were performed with an excitation and collection an angle of 26˚.

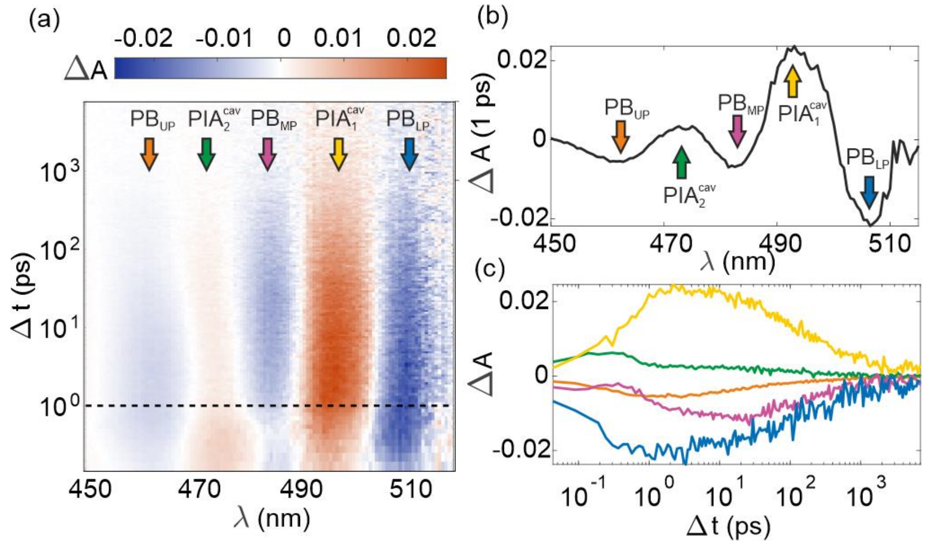

Figure S9. **Complementary ultrafast transient absorption spectroscopy maps and dynamics of a $CsPbBr_3$-QD cavity.** (a) Transient absorption spectra map obtained employing a different non-linear crystal (CaF_2) and at an angle of incidence of 45º with respect to the sample normal, which allows us to shift the measurable wavelength range to the blue so we can access the information related to the UP. (b) Selected ΔA spectrum (Δt=1 ps). Arrows indicate the spectral position of bleached (blue, pink and orange arrows for PB_{LP}, PB_{MP} and PB_{UP}, respectively) and photoinduced absorption (yellow and green arrows for PIA_1^{cav} and PIA_2^{cav}, respectively) signals. (c) Time evolution of the maximum/minimum intensities of the main signals extracted from the analysis of ΔA. The dynamics of the UP bleaching (PB_{UP}, occurring at λ≈460 nm and highlighted by an orange arrow in Fig.S9b) follows a similar trend than that observed for the other two bleaching signals (PB_{MP} and PB_{LP}, occurring at λ≈485 nm and λ≈510 nm, respectively and pointed out by pink and blue arrows in Fig.R3b), but with a shorter lifetime than that of the PB_{MP} and PB_{LP}, as expected for this higher energy polariton state. Accordingly, the corresponding PIA_2^{cav} (λ≈470 nm, green arrow in Fig.R3b) shows also a much shorter lifetime than PIA_1^{cav} (λ≈495 nm, yellow arrow in Fig.R3b), as well as a much less pronounced intensity.

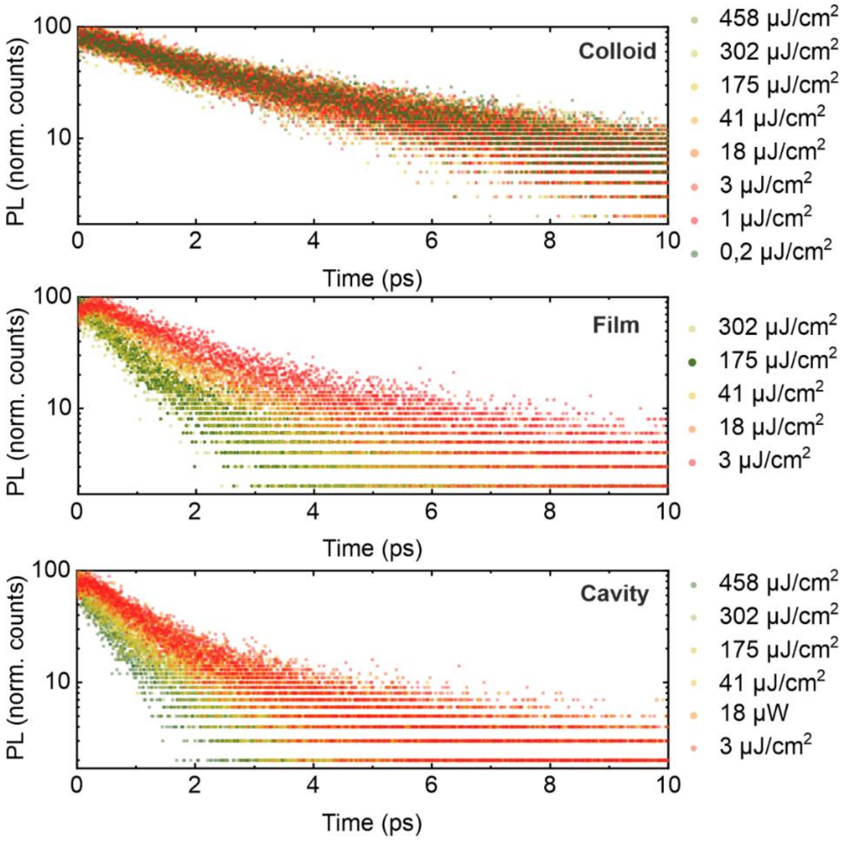

Figure S10. Photoluminescence vs. time of the three systems (colloid, film and cavity) at different fluencies with λ_{exc}=420nm.

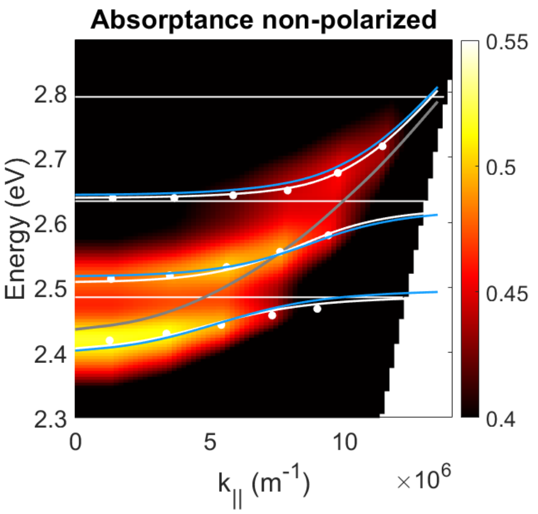

Figure S11. Calculated dispersion relations attained considering different oscillator strengths. The intensity map shows the experimental absorption energy dispersion relation. Superimposed, the energy dispersion relations attained from the solution of the three coupled oscillator Hamiltonian (Eq. S4) using the initially estimated input parameters ($\hbar\Omega_{\gamma,s}$=87 meV/f_s=0.85 and $\hbar\Omega_{\gamma,p}$=77 meV/f_p=0.66, blue lines) and those attained for the optimum fit ($\hbar\Omega_{\gamma,s}$=81.7 meV/f_s=0.75 and $\hbar\Omega_{\gamma,p}$=64.2 meV/f_p=0.46, white lines).

Supplementary Methods

1. Refractive index and reflectance of the underlying cavity.

The effective refractive index used to calculate the underlying cavity modes, i.e. those that would be observed if there was a non-absorbing layer between the mirrors, is estimated from the experimentally estimated n and k curves shown in Figure S2. From this, we set 1.84 as constant refractive index for the non-absorbing cavity, as it is the average value attained at the spectral range at which the polaritonic modes are observed. Also, in all cavity reflectance/absorptance calculations, the variations of the PQD layer thickness observed by electron microscopy are accounted for by averaging a gaussian distribution centered at 356 nm with a 25 nm FWHM.

2. Tavis-Cummings Hamiltonian solutions: polaritons, dark states and Hopfield coefficients.

Within the single excitation subspace, strong coupling between *N* electronic transitions and one photon resonant mode leads to the formation of *N*+1 coherent superpositions of excitations. Since the cavity dominates the response under external driving, and having the lower and upper hybrid light-matter states the highest contribution of the cavity mode, the absorption spectrum of the strongly coupled system shows mainly two peaks, corresponding to the lower and upper polaritons. The other *N*−1 levels (i.e., the vast majority of solutions), whose energies lie in between those of the upper and lower polaritons, cannot be accessed from the ground state for symmetry reasons and are referred to as dark states. In our case, we have *N* $CsPbBr_3$-QDs and two excitonic transitions per PQD, $\hbar\omega_s$ and $\hbar\omega_p$, involved in the coupling, which yields 2*N*+1 coherent superpositions of excitations, of which 2*N*-2 are **dark states** and 3 are the lower, middle and upper polaritons. With this in mind, we can obtain relevant information on the superposition of states leading to the formation of these polaritons by solving a simplified eigenvalue equation for the Tavis-Cummings Hamiltonian, $\hat{H}_{TC}$:

$$\hat{H}_{TC}\psi_{L,M,U} = \hbar\omega_{L,M,U}\,\psi_{L,M,U} \quad \text{(S1)}$$

The new hybrid states, ψ_L, ψ_M, and ψ_U, are the wavefunctions of the LP, MP and UP states, which result from the hybridization of the cavity photon and the two excitonic transitions involved in the coupling, described by the wavefunctions $\psi_\gamma, \psi_s, \psi_p$:

$$\psi_{L,M,U} = C_\gamma^{L,M,U}\psi_\gamma + C_s^{L,M,U}\psi_s + C_p^{L,M,U}\psi_p \text{(S2)}$$

In equation (S2), C_i^j are the **Hopfield coefficients**, which fulfill the normalization condition:

$$\left|C_\gamma^j\right|^2 + \left|C_s^j\right|^2 + \left|C_p^j\right|^2 = 1 \qquad \text{(S3)}$$

On this grounds, Eq.(S1) becomes:

$$\begin{pmatrix} \hbar\omega_\gamma & \hbar\frac{\Omega_{\gamma,s}}{2} & \hbar\frac{\Omega_{\gamma,p}}{2} \\ \hbar\frac{\Omega_{\gamma,s}}{2} & \hbar\omega_s & 0 \\ \hbar\frac{\Omega_{\gamma,p}}{2} & 0 & \hbar\omega_p \end{pmatrix} \begin{pmatrix} C_\gamma \\ C_s \\ C_p \end{pmatrix} = \hbar\omega_{L,M,U} \begin{pmatrix} C_\gamma \\ C_s \\ C_p \end{pmatrix} \qquad \text{(S4)}$$

By solving this three coupled oscillator Hamiltonian, we attain the polariton energy dispersion curves, $\hbar\omega_{L,M,U}$ vs. $\mathbf{k}_{\|}$, which are plotted as white solid lines in Fig. 2a-2b in the main body of the manuscript, and the the Hopfield coefficients $\left|C_i^j\right|^2$, which give us the degree of contribution of each state to the different exciton-polariton states observed, as shown also in Figs. 2c-2e. Please note that in Eq.(S4), the number of identical excitonic transitions, N, coupled to the optical mode is included inside the coupling (off-diagonal) terms, through the Rabi frequencies $\Omega_{\gamma,s} = \sqrt{N_s} g_{\gamma,s}$, and $\Omega_{\gamma,p} = \sqrt{N_p} g_{\gamma,p}$, where g is the coupling strength of that particular transition. ω_{exc} and ω_{mode} represent the exciton and photonic mode original frequencies. It should be noted that the description in terms of N particles and 1 mode is only a fair approximation to describe a planar cavity with a continuum of in-plane wave vectors k and the associated dispersion (i.e., the angle-dependent frequency). A more rigorous discussion of these systems may be made in terms of the density of states of molecular vs cavity excitations, as, e.g., in ref.2.

In order to solve the eigenvalue equation S4, and hence attain the dispersion relation and the Hopfield coefficients plotted in Fig. 2, we have to introduce the off-diagonal terms $\hbar\Omega_{\gamma,s}$ and $\hbar\Omega_{\gamma,s}$ (i.e., the coupling parameters) in the corresponding three-coupled oscillator Hamiltonian. These are given by:

$$\hbar\Omega_{\gamma,i} = \sqrt{\frac{3e^2\hbar^2 f_i N_{QD}}{m_e \varepsilon_0 V_C}} \qquad \text{(R.1)}$$

, where e and m_e are the electron charge and mass, respectively, $\hbar$ is the reduced Planck constant, ε_0 is the permittivity of vacuum, f_i is the oscillator strength of the transition (1s-1s for i=s or 1p-1p for i=p), N_{QD} is the number of QDs participating in the coupling and V_C is the effective cavity mode volume. Considering that the perovskite nanocrystals are uniformly distributed in the film, which implies that the number of QDs contributing to the coupling (N_{QD}) is directly proportional to the effective modal cavity volume (V_C), the ratio N/V_c was approximated by the QD number density, 2.159 x 10^{17} QDs/cm^{-3}. The oscillator strength of the 1s-1s transition was estimated applying the formula:

$$f_s = \frac{6\pi m_e \epsilon_0 c^3}{e^2 n\, \omega}\Gamma_{rad} \qquad \text{(R.2)}$$

, where n is the refractive index of the medium in which the emitter is placed (which we consider to be n≈1.84, according to Fig. S3), c is the speed vacuum speed of light and Γ_{rad} is the radiative decay rate estimated from the time resolved photoluminescence measurements of the QD solid and its PLQY (32%). This formula has been successfully used by Leistikow et al.[3] to perform a systematic analysis of the size-dependent oscillator strength was carried out for a large number of QDs. Within this approach, we obtain f_s=0.85. Substituting the estimated f_s and QD number density in Eq. R1 yields $\hbar\Omega_{\gamma,s}$=87 meV, which is the same value we extract for $\hbar\Omega_{R1}$ from the analysis of the experimental absorption dispersion relation. Since equation R.2 cannot be applied to higher energy transitions, and encouraged by the good agreement between the estimated $\hbar\Omega_{\gamma,s}$ and $\hbar\Omega_{R1}$, we opted for estimating f_p assuming that $\hbar\Omega_{\gamma,p} =$

$\hbar\Omega_{R2}\approx$77meV which gives f_p=0.66. The dispersion relation attained with these values is plotted in Fig. S11 (blue lines). Then, these parameters were gradually varied to attain a best fit to the experiment (white lines), which occurs for $\hbar\Omega_{\gamma,s}$=82 meV (f_s=0.75) and $\hbar\Omega_{\gamma,s}$ =64 meV (f_p=0.46). Hopfield coefficients shown in Fig. 2 in the manuscript were estimated using these optimized values.

3 Comparative analysis of transient absorption spectroscopy results for PQD colloidal dispersions, thin films and optical cavities.

Polariton excitation and decay dynamics in $CsPbBr_3$-QDs optical microcavities were studied by ultrafast transient absorption spectroscopy (TAS) using a rare-earth based femtosecond pulsed laser (pulse duration, 190 fs), whose beams were redirected through either an optical parameter amplifier, to generate the pump pulses, or a delay line and a sapphire crystal, to create broadband probe pulses. For the sake of comparison, a similar analysis was performed for the $CsPbBr_3$-QDs colloidal dispersion and the bare film. Considering the different absorptance of the three systems under analysis, the excitation photon fluence was varied in the range 12-118 $\mu J/cm^2$ to achieve less than one excitation per QD, preventing the damage of the samples. Results are shown in Fig. S6, in which the full series of ΔA spectra (with ΔA being the result of subtracting the linear absorptance from that of the photoexcited sample) attained over a five order of magnitude time scale are plotted as intensity maps as a function of probe photon wavelength and pump-probe delay, Δt, following the non-resonant excitation (i.e., matching nor the excitonic transitions observed in $CsPbBr_3$-QDs colloidal dispersion and film, neither the polaritonic transitions in the cavity) at time 0 by a λ=420 nm pump pulse. Results attained for the PQD colloid, film and optical cavity are plotted in Figs. S6a, S6b and S6c, respectively. Selected ΔA spectra attained at different delay times are explicitly shown in Figs. S6d-S6f, namely at Δt=0.4 ps (green line) and Δt=10 ps (purple line). The early-time dynamics of the main signals identified in Figs. S6a-S6c are plotted in Figs. S6g-S6i, respectively.

The ultrafast response of both the $CsPbBr_3$-QDs colloidal dispersion and film show a prominent photobleaching of the first excitonic transition (PB_{exc}^{col} and PB_{exc}^{film}, centred at 2.48 eV, shaded in blue) and a conspicuous photoinduced absorption (PIA_1^{col} and PIA_1^{film}centred at 2.56 eV, shaded in yellow) spectrally located in between the first and second excitonic transitions, as the comparison between linear absorptance (dashed line) and TAS curves (green and purple solid lines) in Figs. S6d and S6e explicitly reveals. A third less intense and short-lived (<2 ps) signal ($PIA_2^{col}$and PIA_2^{film}, located around 2.40 eV, shaded in orange) is observed at energies right below the first excitonic transition. The PB_{exc} signal is typical of semiconductor nanocrystals and is understood as the result of the filling of the lowest energy excited states as a consequence of the cooling of photocarriers.[4] The short-lived PIA_2 signal, also characteristic of QDs, is usually attributed to a slight red-shift of the 1s-1s transition caused by the Coulomb interaction between photoexcited e^--h^+ pairs, the so-called biexciton effect.[5-7] As cooling takes place, hot carriers decay and fill in these newly available low energy states, thus extinguishing the PIA_2 signal.[8,9] The origin of the intense PIA_1 signal observed in $CsPbBr_3$-QDs, however, has been the subject of debate.[10,] It has been described as a distinctive feature of lead halide PQDs, and attributed to a parity-forbidden transition,[12] only observable due to the relaxation of the selection rules resulting from the formation of large polarons, i.e., significant exciton-induced deformations of the $[PbBr_3]^-$ sublattice.[13,14] In this context, it has been proposed that large polarons in $CsPbBr_3$-QDs could be also contributing to the screening of the Coulomb

interaction between e^{-}-h^{+} pairs,[15] as the coincident dynamics of the PIA_2 decay (orange lines in Figs. S6g and S6h) and of the PIA_1 rise (yellow lines) seem to indicate. On the other hand, recent studies alternatively assign the PIA_1 signal to higher biexcitonic transitions,[16] a hypothesis supported by detailed calculations of the spectrum of confined exciton-to-biexciton transitions in $CsPbBr_3$ lattices. In either case, the PIA_1 signal is considered to be the result of transitions that become available only upon photoexcitation.

Bearing in mind the response of the bare $CsPbBr_3$-QD film, the ultrafast ΔA signal from the optical cavity displayed in Fig. S6c was analysed. Two intense bleaching signals are detected at the lower (PB_{LP}) and middle (PB_{MP}) polariton spectral positions (located at 2.43 eV and 2.55 eV, respectively). Also, a very intense photoinduced absorption (PIA_1^{cav}, at 2.49 eV) is observed between PB_{LP} and PB_{MP}. The early stage dynamics of these signals are compared in Fig. S6i. In there, it can be seen that PB_{LP} and PB_{MP} signals (blue and pink line respectively) present a very different rise time ($\tau_{PB,LP}$<0.5 ps, $\tau_{PB,MP}$<2 ps). Although the bleaching of the lower polariton is the consequence of the characteristic filling of the lowest energy states due to hot-carrier relaxation, the subsequent long-lived bleaching of the middle polariton cannot be understood without considering the interplay with the abovementioned large reservoir of dark states. Even though not directly accessible from the ground state, dark states can be filled both from the lower polariton state and from higher energy levels as hot electrons cool down.[17] This picture is further supported by the partial recovery of the absorption evidenced by the peak observed in the PB_{LP} signal at Δt=0.45 ps (signposted by a vertical grey dashed line in Fig. S6i), which points at a transfer of carriers from the lower polariton state to the dark state reservoir once a certain occupation level is attained. Thus, gradual filling of the dark states could eventually lead to transfer of carriers to the middle polariton state, giving rise to its bleaching. These carrier exchanges are favoured by the significant overlap between dark and polaritonic states, which is expected in our case due to the moderate separation observed between lower and middle polaritons.[18,19] Interestingly, the low energy photoinduced absorption observed in the dispersion and the film, PIA_2, attributed to the shift of the lowest excited state energy caused by biexciton interaction, is almost imperceptible in the cavity, which indicates that no significant renormalization is taking place in the cavity.

4 Transient absorption data treatment.

To analyse the time evolution of the cavity absorptance at resonance with 1s-1s exciton (at 26°) both the spectra of ΔA measured with ultrafast absorption techniques and the linear absorption modified by ΔA were separated in their main components. ΔA stands for the logarithmic ratio of change in absorption after the pump pulse. As the cavity transmittance is negligible in the spectrum range examined, the ΔA is measured by only considering the reflectance:

$$\Delta A = -\log(\frac{R_{exc}}{R_0}) \qquad (7)$$

Where R_0 is the reflectance of reference (pump off) and R_{exc} is the reflectance of the photoexcited cavity at a given Δt (note that for the transmitting samples i.e. the colloidal dispersion and the film the reflectance is negligible and the signal is detected in transmission, redefining ΔA as $\Delta A = -\log(\frac{T_{exc}}{T_0})$). The linear absorptance at each Δt after excitation is then reconstructed from the reflectance attained from the sample in its ground state and the transient absorption spectrum as:

$$A_{exc} = 1 - R_{exc} = 1 - R_0 \cdot 10^{-\Delta A} \qquad (8)$$

Also, in order to analyse the different contributions observed in the ΔA spectra, they were decomposed into Voigt functions, whose parameters were obtained with the Matlab fmincon optimization function. These functions are depicted in Fig. 3f in the main body of the manuscript for the $CsPbBr_3$-QD cavity ΔA attained at Δt=10 ps.

5 Charge carrier density estimation in TAS measurements.

The average value of the pump power (P) is measured at the sample position and it is used to calculate the fluence (fl) of a single pulse:

$$fl = \frac{P}{f \cdot \pi r^2} \qquad (5)$$

Where $f = 1kHz$ is the frequency of the pump pulses and $r = 100\ \mu m$ is the radius of the spot illuminating the sample.

The density of charge carriers per volume unit (η_{exc}) generated by each pump pulse, after correcting by the sample reflection, is calculated using the formula described by Savill et. al.[20] that considers a mean density inside the sample calculating the average between the charge carriers that would be generated at the top surface of the sample and the ones at the end of the sample after the absorption of a fraction of the incoming photons:

$$\eta_{exc} = \frac{fl(1-e^{-\alpha \cdot d \cdot ff})}{d \cdot ff \cdot E_{photon}} \qquad (6)$$

being α the absorption coefficient of the QDs, d the thickness of the sample, ff the filling fraction or the fraction of QDs volume in the sample (assuming the whole volume is the sum of ligands, polystyrene and QDs) and $E_{photon}$the energy of one photon (in our case, that corresponding to a pump wavelength of 420nm).This quantity multiplied by the volume of a nanocrystal, which it is assumed to be a sphere with a 6.6nm radius, gives us the charge carrier density per PQD, N_{exc}.

6 Log-normal fit of PL decay curves.

PL decay curves were fitted using an exponential model weighted by with a gaussian distribution of decay rates (log-normal function)[21]. PL counts at each time are then given by

$$\int_0^{\infty} \rho(\Gamma) \exp(-\Gamma t) d\Gamma \qquad (9)$$

where Γ is the decay rate and $\rho(\Gamma)$ is the gaussian distribution of decay rates:

$$\rho(\Gamma) = C \frac{1}{\sigma \Gamma \sqrt{2\pi}} \exp\left[-\frac{1}{2}\left(\frac{ln\Gamma - \mu}{\sigma}\right)^2\right] \qquad (10)$$

Here, C is an amplitude factor, σ is the standard deviation and μ the mean of the logarithm of Γ. Fitting to experimental data was performed using Matlab non-linear regression function *Nlinfit* to estimate the parameters *C*, μ and σ with the model described in expressions (9) and (10).

	μ	σ	C	Γ	FWHM	t_{max} (ns)
PQD Cavity	0.1659504	0.701809	119.98	0.721383	1.796402	**1.35**
Bare PQD film	-0.534556	0.527388	108.05	0.443661	0.6470751	**2.22**
PQD solution	-1.10507	0.232735	78.705	0.313725	0.155552	**3.23**

Table S1. Values of the parameters attained for the optimum fittings of the time resolved PL measurements using Eq. 10.

[1] Manser, J.S.; Kamat, P.V. Band filling with free charge carriers in organometal halide perovskites, *Nat. Photon.* **8**, 737-743 (2014).

[2] del Pino, J., Feist, J., García-Vidal, F.J. Quantum theory of collective strong coupling of molecular vibrations with a microcavity mode. *New J. Phys.* **17**, 053040 (2015)

[3] Leistikow, M. D. et al. Size-dependent oscillator strength and quantum efficiency of CdSe quantum dots controlled via the local density of states. *Phys. Rev. B* **79**, 045301 (2009)

[4] Klimov, V. I. Spectral and Dynamical Properties of Multiexcitons in Semiconductor Nanocrystals. *Annu. Rev. Phys. Chem.* **58**, 635–73 (2007)

[5] Klimov, V., Hunsche, S. & Kurtz, H. Biexciton effects in femtosecond nonlinear transmission of semiconductor quantum dots. *Physical Review B* **50**, 8110 (1994)

[6] Klimov, V. I. Optical Nonlinearities and Ultrafast Carrier Dynamics in Semiconductor Nanocrystals. *J. Phys. Chem. B* **104**, 6112-6123 (2000)

[7] Makarov, N. *et al.* Spectral and Dynamical Properties of Single Excitons, Biexcitons, and Trions in Cesium−Lead-Halide Perovskite Quantum Dots. *Nano Lett.* **16**, 2349−2362 (2016)

[8] Price, M. B. *et al.* Hot-carrier cooling and photoinduced refractive index changes in organic–inorganic lead halide perovskites. *Nature Communications* **6**, 8420 (2015)

[9] Manser, J. S. & Kamat, P. V. Band filling with free charge carriers in organometal halide perovskites. *Nature Photonics* **8**, 737–743 (2014)

[10] Barfüßer, A. *et al.* Confined Excitons in Spherical-Like Halide Perovskite Quantum Dots. *Nano Lett.* **22**,8810−8817 (2022)

[11] Piatkowski, P. *et al.* Direct monitoring of ultrafast electron and hole dynamics in perovskite solar cells. *Phys. Chem. Chem. Phys.* **17**, 14674−14684 (2015)

[12] Rossi, D. *et al.* Light-Induced Activation of Forbidden Exciton Transition in Strongly Confined Perovskite Quantum Dots. *ACS Nano* **12**, 12436−12443 (2018)

[13] Zhu, H. *et al.* Screening in crystalline liquids protects energetic carriers in hybrid perovskites. *Science* **353**, 1409-1413 (2016)

[14] Seiler, H. *et al.* Direct Observation of Ultrafast Lattice Distortions during Exciton−Polaron Formation in Lead Halide Perovskite Nanocrystals. *ACS Nano* **17**, 1979−1988 (2023)

[15] Akkerman, Q. et al. Controlling the nucleation and growth kinetics of lead halide perovskite quantum dots. Science 377, 1406–1412 (2022)

[16] Schmitt-Rink, S., Chemla, D. S. & Miller, D. A. B. Theory of transient excitonic optical nonlinearities in semiconductor quantum-well structures. Phys. Rev. B 32, 6601 (1985)

[17] Gonzalez-Ballestero, C., Feist, J., Badía, E. G., Moreno, E. & García-Vidal, F. J. Uncoupled Dark States Can Inherit Polaritonic Properties. *PRL* **117**, 156402 (2016)

[18] Groenhof, G. Climent, C. Feist, J. Morozov, D. & Toppari, J. J. Tracking Polariton Relaxation with Multiscale Molecular Dynamics Simulations. *J. Phys. Chem. Lett.* **10**, 5476−5483 (2019)

[19] Xiang, B. *et al.* State-Selective Polariton to Dark State Relaxation Dynamics. *J. Phys. Chem. A* **123**, 5918−5927 (2019)

[20] Savill, K. J., Klug, M. T., Milot, R. L., Snaith, H. J., & Herz, L. M. Charge-carrier cooling and polarization memory loss in formamidinium tin triiodide. *J. Phys. Chem. Lett*. **10,** 6038-6047 (2019).

[21] Ibisate M., Galisteo-López J.F., Esteso V. & López C. FRET-Mediated Amplified Spontaneous Emission in DNA-CTMA complexes, *Adv. Opt. Mat.* **1**, 351-656 (2013).
